# Physical fitness levels among children in northeast Italy by sex and age group: a comparison with teachers’ assessments and children in other European countries

**DOI:** 10.3389/fspor.2024.1383575

**Published:** 2024-12-03

**Authors:** Andrea Toscani, Arve Vorland Pedersen

**Affiliations:** Department of Neuromedicine and Movement Science, Faculty of Medicine and Health Sciences, Norwegian University of Science and Technology, Trondheim, Norway

**Keywords:** pupil, adolescent, field test, motor coordination, motor development, function

## Abstract

**Introduction:**

Physical fitness is associated with health-related quality of life, especially among youth. Although schools play an important role in promoting children's physical activity, in Italy the lack of qualified physical education teachers in primary schools may be compromising children's achievement of recommended levels of physical activity.

**Methods:**

To test that possibility, we measured the physical fitness of 170 children (i.e., 79 boys and 91 girls) 6-10 years old in two schools in Cadore, Veneto, in northeast Italy, using the Physical Fitness Test, a tool developed in Norway and previously used among children there and in Lithuania. Teachers in Italy also assessed their students' physical fitness, and their rankings were correlated with the children's test results. The test battery included nine elements: a standing broad jump, hopping 7 m on both feet, hopping 7 m on one foot, throwing a tennis ball, pushing a medicine ball, climbing wall bars, a 10 × 5 m shuttle run, a 20 m run, and a 6 min Cooper test.

**Results:**

Test scores generally increased with age and more steeply among boys than girls, and boys outperformed girls on most items. Children in Italy performed similarly to children in Norway but outperformed ones in Lithuania on nearly every item across sex and age groups. The correlation between teachers' predictions and the actual test results was rather low for boys (.538, *p* < .001) and even lower for girls (.360, *p* < .001).

**Discussion:**

Data revealed similar results in physical fitness between the three countries, albeit with some differences for individual items. However, primary school teachers in Italy assessed physical fitness rather poorly, possibly due to their lack of specific academic training in physical education and thus limited perception of physical fitness compared with academically trained physical education teachers. Last, because the procedure for categorizing children by age may significantly affect the results and subsequent between-group comparisons, researchers comparing children in different age groups should report their procedures for categorizing age.

## 1 Introduction

Physical fitness (PF) is important for maintaining good health and preventing early death from a range of diseases (1). PF stems from physical activity (PA) (2), which partly explains why the World Health Organization (WHO) and national health authorities in most countries have launched strategies for increasing PA in populations worldwide (3). Children's PA is particularly important due to its short-term effects on PF, and evidence shows that individuals who are physically active as children are more likely to maintain their PA level into adulthood (4). In fact, enhancing PF levels among children has been found to reduce the risk of not only overweight and obesity throughout puberty (5) but also noncommunicable diseases later in life, including cardiovascular disease, cancer, diabetes, and various chronic respiratory diseases (3).

By comparison, low PF levels are related to *vulnerability*, defined as “a dynamic process of stress and resources across various domains of life, levels, and time” (6), and relatively poor academic outcomes (7). Beyond that, a sufficient level of PF is considered to be important for children's development and maintenance of physical, physiological, psychomotor, and psychosocial functions (8), while a high level of cardiorespiratory fitness is associated with better mental and skeletal health (9).

Although PF is partly predetermined by genetic factors, there are vast possibilities for change depending on the level of regular PA (2). According to the WHO's recommendations, a daily average of 60 min of moderate to vigorous PA, mostly aerobic, among 5–17-year-olds is associated with better physical, mental, and cognitive health (10). Moreover, data have shown that more time spent in moderate to vigorous aerobic PA and muscle-strengthening activities can increase cardiorespiratory fitness and muscular fitness, respectively, with additional evidence showing adjunctive benefits that result in a combination of both. By contrast, according to the same review, an association exists between time spent sedentary and adverse health outcomes (10). For adults, suggested levels of PA are communicated as weekly volumes of aerobic and strengthening activity, for a recommended average of 150–300 min of moderate PA, 75–150 min of vigorous PA, or a combination of the two. Aside from aerobic PA, 2 or more days of moderate or vigorous muscle-strengthening PA per week can provide additional health benefits (10). Thus, the primary differences between recommendations for children and for adults concern the total volume, intensity, and type of PA, and although the WHO recommends that children engage in PA every day, its guidelines do not specify the recommended intensity of PA.

However, at odds with PA and thus PF, modern society continues to facilitate sedentary lifestyles in work, play, and even transportation (11). Indeed, a recent systematic review revealed a decrease in cardiorespiratory endurance in the general human population, particularly from 1986 to 2012 (12), and in 2016, 80% of children and adolescents worldwide did not fulfill the mentioned WHO guideline of 60 min of moderate to vigorous PA per day (13). Furthermore, in a study assessing sedentary time and levels of light, moderate, and vigorous PA among 686 boys and girls 10–12 years old in five European countries (i.e., Belgium, Greece, Hungary, the Netherlands, and Switzerland), girls were found to spend significantly more time sedentary and significantly less time in light or moderate to vigorous PA than boys (14). By sex and country, the most sedentary children were boys in Greece and girls in the Netherlands, and overall, only 4.6% of the girls and 16.8% of the boys met the WHO's recommendations. Even though children in countries such as Finland and Canada have unexpectedly shown improved strength and cardiorespiratory levels in other studies, those findings do not shift the general trend (2).

Meanwhile, in Italy, the EpiCentro Indagine 2019, an annual survey conducted by the Italian National Institute of Health, revealed that 20.4% and 9.4% of children aged 7–8 years were respectively overweight and obese (15). Moreover, in 2006, children in Italy ranked among the worst performers on the Léger test (16)—a 20 m shuttle running test that is commonly used to measure cardiorespiratory health (17)—whereas children in Northern European countries achieved the best results (18).

To combat those negative trends, schools could play a more active role in promoting an environment that facilitates PA and thereby helps children to meet recommended levels for PA (19). However, doing so requires physical education (PE) teachers to possess sufficient knowledge about PA, as well as about how to effect changes in the PA of their students. Unlike in many European countries—for example, Spain, Belgium, Latvia, and Greece (20)—such criteria have not always been met in Italy's primary schools, where teachers need no specific qualifications to teach PE (21). Against that trend, the Italian government recently enacted new regulations in primary schools that require any instructor who teaches PE to possess formal PE education. Introduced in the 2022–2023 academic year and initially taking force in the fifth grade (i.e., 9–10 years old), the regulation marks a rather significant step toward increasing children's awareness about the importance of PA and thus motivating them to be physically active (22). However, the development has raised questions about whether the current generalist teachers possess the required knowledge about PA and about teaching PE, as well as whether specialist teachers with a background in sports and professionals educated in sports science possess the skills needed to interact with children or the didactic skills needed to teach PE (21).

Aside from planning and teaching, which represent the ability to effectively organize and engage students, a third factor to be included in the PE teacher's professional tool kit is the ability to assess children's performance (23). In fact, the authors argued, the skills required for evaluating children in PE at school include a combination of both theoretical and practical knowledge in the field being of assessed and the ability to administer test batteries for PF and motor skills.

In past decades, the many tests for PF, mostly developed for adults, may have compromised the results obtained for children as well as complicated comparisons between groups such as national populations (24). Even more problematic, those tests often require sophisticated machinery and equipment that is usually available only in specialized laboratories. As a consequence, it has been difficult to provide sufficient data and conduct longitudinal studies. For those reasons, Fjørtoft et al. developed a simple test suitable for children 5–12 years old that is easy to administer and aimed at providing reliable, objective, quantitative data about PF (25). To date, the test has been used in Norway (25–29), Lithuania (30), and Iceland (31), while individual items retrieved from it have been used in other countries, including Italy (32–34). The test involves performing everyday functional tasks, assessed in terms of strength, endurance, motor coordination, balance, and agility. Furthermore, by including both the upper and lower body, the test considers all physical functionality (18).

In their validation of the test, Fjørtoft et al. asked an experienced PE teacher to rank the children in his class according to his perception of their PF and correlated the teacher's assessment with the children's test scores (25). The very high correlation indicated that the test could not only measure PF according to a construct similar to the one undergirding the teacher's assessments but also allowed observing and assessing the children's PF such that it corresponded with test results. Thus, the same strategy could be used to assess the ability of other teachers to perform similar assessments.

Considering all of the above, we wanted to measure the PF of children in Italy with Fjørtoft et al.'s (25) test to determine whether it would generate results similar to what previous studies have shown using the same test. We also wanted to determine whether the test results would correlate with teachers’ assessments of their students’ PF and thus whether the teachers’ perception of PF matched the perception measured by the test. If so, then the easy-to-use test could be used to compare children's PF between schools in Italy and to make cross-national comparisons when the same test has been used. Beyond that, the test results could aid teachers in Italy in assessing their students’ PF. At the same time, although some studies have used the mentioned test, because none of them investigated children in Italy, we did not know what results to expect. Moreover, those studies had diverse research questions and differed from each other and our study in many ways. Thus, our study's design was exploratory, no specific hypotheses were tested, and as in previous similar studies, we sought to include a similar number of children.

## 2 Materials and methods

### 2.1 Participants

Children in 14 classes from two primary schools in Cadore, Veneto, in northeastern Italy participated in our study. Permission was formally obtained from both schools’ principals, and teachers were informed about the study's procedure. Written informed consent was obtained from parents based on information about the benefits and risks of participating in the study. Of the 184 children whose parents provided consent, 170 children (i.e., 79 boys and 91 girls) who met the inclusion criteria (i.e., having no particular diseases and being able to perform the test) were included in the study. As for the 14 others, 11 children were absent from school on the test day, one withdrew from the test, and two were excluded due to being outside the age range. The age groups ranged from 6 to 10 years—that is, approximately from the first to fifth grade (*M* = 8.69, *SD* = 1.5). Table 1 shows the distribution of the children by age group, with the mean (*M*) and standard deviation (*SD*) of the age on test day (i.e., relative age) and anthropometric measures.

**Table 1:** Mean (*M*) and standard deviation (*SD*) of age on test day and anthropometric measures by age group.

Age group	Sex		Age at test		Height (m)		Weight (kg)		Body mass index	
	Male (*n*)	Female (*n*)	*M*	*SD*	*M*	*SD*	*M*	*SD*	*M*	*SD*
6	12	29	6.70	0.30	1.20	0.06	23.6	5.1	16.2	2.4
7	16	14	7.73	0.27	1.26	0.06	26.9	6.0	16.7	2.8
8	13	13	8.78	0.27	1.34	0.06	32.1	6.5	17.7	3.0
9	24	18	9.77	0.28	1.39	0.07	34.4	7.1	17.8	2.8
10	14	17	10.75	0.30	1.47	0.08	44.2	12.1	20.3	4.6

The study was conducted in accordance with the Declaration of Helsinki and approved by the Norwegian Regional Research Ethics Committee (Application No. 381916) on November 24, 2021.

The Norwegian PF test involves nine items (25): jumping as far as possible with both feet (i.e., measured in cm), hopping 7 m on two feet as fast as possible (i.e., measured in s); hopping 7 m on one foot as fast as possible (i.e., measured in s); throwing a tennis ball with one hand as far as possible (i.e., measured in m); pushing a 1 kg medicine ball with both hands as far as possible (i.e., measured in m); climbing up, crossing, and climbing down wall bars as fast as possible (i.e., measured in s); performing a 5 m shuttle run 10 times as fast as possible (i.e., measured in s); and a 6 min Cooper test (i.e., measured in m). Each child's age (d/m/y), sex (b/g), height (m), and weight (kg) were also collected as prescribed in the test manual (25). The materials used during data collection were a digital stopwatch, a digital scale, a measuring tape, masking tape, cones, gym mats, four-column wall bars, a 1 kg medicine ball, and a tennis ball. Because one of the schools was not equipped with wall bars, 117 children did not perform the wall-bar climbing task. Altogether, the nine test items measure a range of different aspects of PF, including power, strength, and endurance; thus, intraclass correlations between items range from.31 to.85 (25). However, correlations between individual items and the total score are much higher and range between.65 and.85 (25). Moreover, because the test aims to measure functional PF and present test items that are familiar to children and easy to understand, the test seems to measure a construct of PF that teachers recognize and perceive as being representative of PF. Last, no expensive equipment is needed, and the test can be administered by individuals without specific education or training (25).

As mentioned, the PF test Fjørtoft et al. (25), which was originally developed in 2003, has since been used in studies conducted in several different countries. The test's items were also sampled from previously published tests and test batteries, which increases the validity. Many of the items have later appeared in still other test batteries, including for example the 20 m shuttle run and the standing long jump, which are used in the more recently developed FitBack program (35). The PF test was chosen because it was simple and needed no expensive equipment. Most importantly, it seems to measure a construct of PF that corresponds well with the perception of PF of practitioners in relevant fields, and it uses test items that are familiar to children. Moreover, in Fjørtoft et al.'s (25) article, the test results of children from one class were compared with their PF as assessed by their PE teacher, who ranked 10 boys and 10 girls according to his perception of their PF and found that the assessments correlated highly with their test scores (i.e.,.93 for girls and .90 for boys). In our study, we wanted to use the same procedure among teachers in Italy to determine how well their assessments correlated with the PF test and thus whether their perception of PF was similar to the perception of the teacher in Norway. In fact, our research question concerning the teachers’ ability to assess children's PF was inspired by Fjørtoft et al.'s article.

### 2.2 Procedure

Participating children were tested during school hours in their school gym or schoolyard during a 1-week period in March. All nine test items were performed on the same day by every child, each of whom was asked in advance to wear comfortable clothing to school. Following the test procedure (25), each item was administered individually, except for the reduced Cooper test, which was performed by the entire class at the same time. After height and weight measurements and before data collection with the test, a 5-minute warm-up session was held, after which each item was explained and demonstrated before measurement was performed, and, aside from the three runs, each participant had two attempts, the best of which was scored. In rare cases in which a child made two procedural errors or could not perform an item, the item was scored as “Failed.” Ultimately, 16 children had a total of 19 failed items out of 1,400 total. As mentioned, many of the children did not perform the wall-bar climbing task because their school had no wall bars. The item was nevertheless included in the overall *z* score, but between-group differences in climbing the wall bars are not discussed in this article.

Teachers were also asked to rank the boys and girls in their class separately based on their perception of each child's overall PF level relative to their peers (i.e., 1st = most physically fit, 2nd = second-most physically fit, etc.), without being provided with any knowledge about the test or the individual items. Those predictions were compared with test results by correlating the two ranks for each class.

Although none of the teachers had formal education in PE, a few had completed short courses, and some may have had other sports-related skills. However, only information about their formal education was collected in our study.

### 2.3 Statistical analyses

Statistical analyses were performed using IBM SPSS version 27.0 for Windows. We used the Kolmogorov–Smirnov test for normality, the two-sample *t* test for normally distributed means comparisons, the Mann–Whitney *U*-test for comparisons of non-parametric means, the Hodges–Lehmann estimator for the confidence interval (CI) of the difference in the distribution of non-parametric means, linear regression analyses, and the Kendall tau-b correlation for ranks. Every element of the test was converted to a *z* score, and to that end, test elements measured as time spent were transformed using the formula *1/score*, such that higher scores always indicated better results. A total score for each child was calculated as the average of the *z* scores for each item on the test successfully completed (25). The normality of distributions was analyzed with the Kolmogorov–Smirnov test and interpretations of histograms, while the presence of outliers and skewed distributions required using non-parametric tests. A *p* value equal to or less than.05 was considered to indicate statistical significance.

Missing data were treated as missing, which reduced the *N* value for the relevant variables. Most missing data were missing due to the lack of wall bars in one of the schools, which prevented testing the wall-bar climbing task; however, because the lack was considered to be a random event, the item was kept in the analyses and is reported in tables but was not used to make any conclusions about between-group differences except as part of the total *z* score due to the smaller *N* value.

## 3 Results

Table 2 shows the *M* and *SD* for each item on the PF test performed by boys and by girls, as well as the *p* value and 95% CI for the mean difference (*MD*). Apart from hopping on one foot, hopping on both feet, and climbing the wall bars, boys performed significantly better on all test items than girls, as detailed in Table 2. Using a non-parametric test was necessary due to some skewed distributions and the presence of outliers.

**Table 2:** Mean (*M*) and standard deviation (*SD*) of test elements for boys and girls with relative *p* value and 95% confidence interval (CI) of the mean difference.

Test item	Total *M*	Total *SD*	Sex	*N*	*M*	*SD*	*p*	95% CI
Standing broad jump (cm)	126.71	21.67	M	79	132.48	23.85	.001	4.39, 17.17
			F	91	121.70	18.28		
Hopping on two feet (s)	3.81	0.77	M	79	3.81	0.87	.614*	−0.28, 1.60**
			F	90	3.81	0.68		
Hopping on one foot (s)	3.44	0.84	M	78	3.46	1.01	.482*	−0.29, 0.14**
			F	90	3.43	0.68		
Throwing a tennis ball (m)	10.63	4.28	M	79	12.31	4.76	<.001	1.93, 4.36
			F	91	9.17	3.19		
Pushing a medicine ball (m)	3.78	1.00	M	79	4.02	1.06	.005*	0.16, 0.74**
			F	91	3.57	0.90		
Climbing wall bars (s)	23.44	16.14	M	27	21.69	13.88	.436*	−8.42, 4.33**
			F	25	25.32	18.37		
10 × 5 m shuttle run (s)	24.02	2.88	M	79	23.70	3.30	.037*	−1.67, −0.6**
			F	91	24.29	2.44		
20 m run (s)	4.64	0.59	M	78	4.56	0.61	.032*	−0.37, −0.2**
			F	91	4.71	0.56		
Reduced Cooper test (m)	828.12	173.08	M	68	896.26	184.26	<.001	68.86, 172.74
			F	88	775.47	144.18		

* Mann–Whitney *U* test for independent non-parametric distributions.

** Hodges–Lehmann estimation of 95% CI.

**Figure 1:**
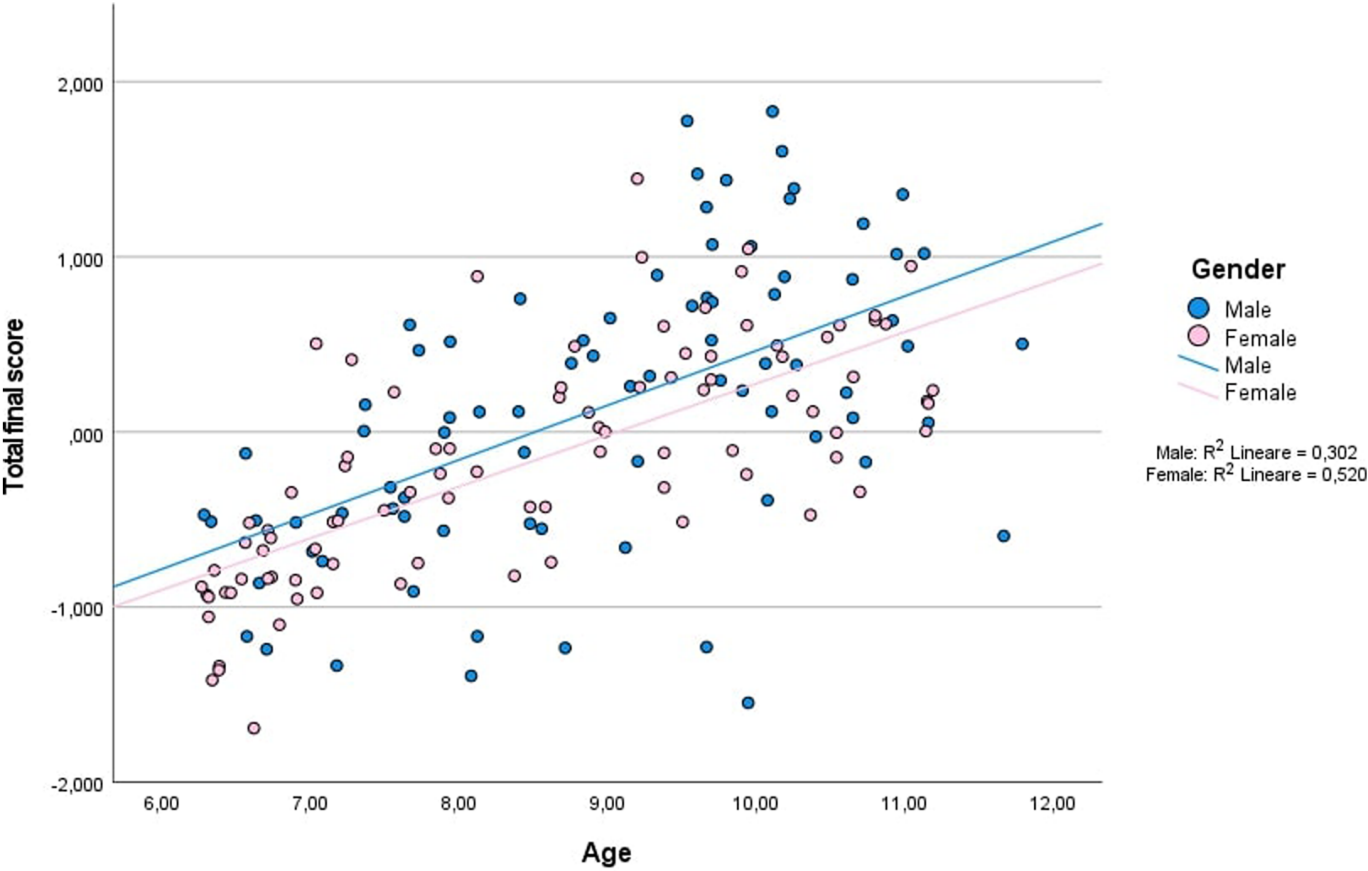
Scatterplot of *z* scores for the overall test across age groups (*N* = 170), with a linear regression representation for boys (*r* = .351) and girls (*r* = .520).

Figure 1 presents a scatterplot of the total *z* scores, which show an increase with age for both boys and girls. Despite some outliers, the total score increased linearly with age and more rapidly for boys than for girls. That trend indicates that older children performed better than younger ones, as aligns with the nature of the test. Whereas 6-year-old boys and girls showed very similar test results, 10-year-old boys achieved significantly higher mean total scores than 10-year-old girls (*p* = .039, 95% CI: −0.695, −0.019). The Kolmogorov–Smirnov test for normality and the relative histogram showed that total scores were normally distributed (*p* > .2).

Next, Table 3 shows results for individual items for both sexes across all age groups. A general increase in scores with age emerged for all test items for boys as well as girls. Statistical tests for comparisons were not viable, however, due to the small number of children in each subgroup.

**Table 3:** Mean (*M*) and standard deviation (*SD*) of test elements across sex and age groups.

Test item	Sex	Age group									
		6		7		8		9		10	
		*M*	*SD*	*M*	*SD*	*M*	*SD*	*M*	*SD*	*M*	*SD*
Standing broad jump (cm)	M	117.25	14.13	125.31	15.36	124.77	26.02	142.46	25.81	143.79	22.59
	F	109.55	15.57	117.93	12.84	122.38	18.68	137.28	10.24	128.53	18.58
Hopping on two feet (s)	M	4.30	0.72	4.02	0.66	3.98	0.95	3.53	1.07	3.49	0.40
	F	4.35	0.56	3.72	0.80	3.72	0.46	3.38	0.52	3.48	0.49
Hopping on one foot (s)	M	4.13	0.69	3.96	1.55	3.49	0.74	2.93	0.66	3.15	0.56
	F	4.03	0.63	3.22	0.47	3.19	0.52	3.05	0.58	3.20	0.43
Throwing a tennis ball (m)	M	7.71	1.63	10.02	2.51	10.95	3.24	15.15	5.58	15.25	3.07
	F	6.06	2.00	9.07	1.78	9.61	2.08	11.48	2.72	11.75	2.41
Pushing a medicine ball (m)	M	2.71	0.45	3.31	0.50	4.18	0.59	4.51	1.02	4.98	0.66
	F	2.71	0.52	3.13	0.66	3.80	0.59	4.41	0.43	4.34	0.59
Climbing wall bars (s)	M	28.52	26.97	34.77	10.50	19.42	6.80	16.77	8.24	12.55	6.00
	F	30.71	13.64	46.31	40.24	24.82	9.64	13.89	7.04	12.57	2.58
10 × 5 m shuttle run (s)	M	26.97	4.25	24.44	2.29	24.32	2.51	22.07	3.07	22.28	1.90
	F	25.85	2.30	24.28	1.94	24.35	2.43	22.91	2.31	23.07	1.71
20 m run (s)	M	5.06	0.31	4.75	0.63	4.59	0.50	4.26	0.70	4.44	0.40
	F	5.19	0.47	4.62	0.30	4.53	0.63	4.27	0.37	4.56	0.42
Reduced Cooper test (m)	M	779.22	131.52	841.50	204.05	885.36	161.42	973.09	198.63	901.15	145.30
	F	709.44	119.09	773.46	98.92	834.15	190.37	803.44	130.98	807.35	158.48

**Figure 2:**
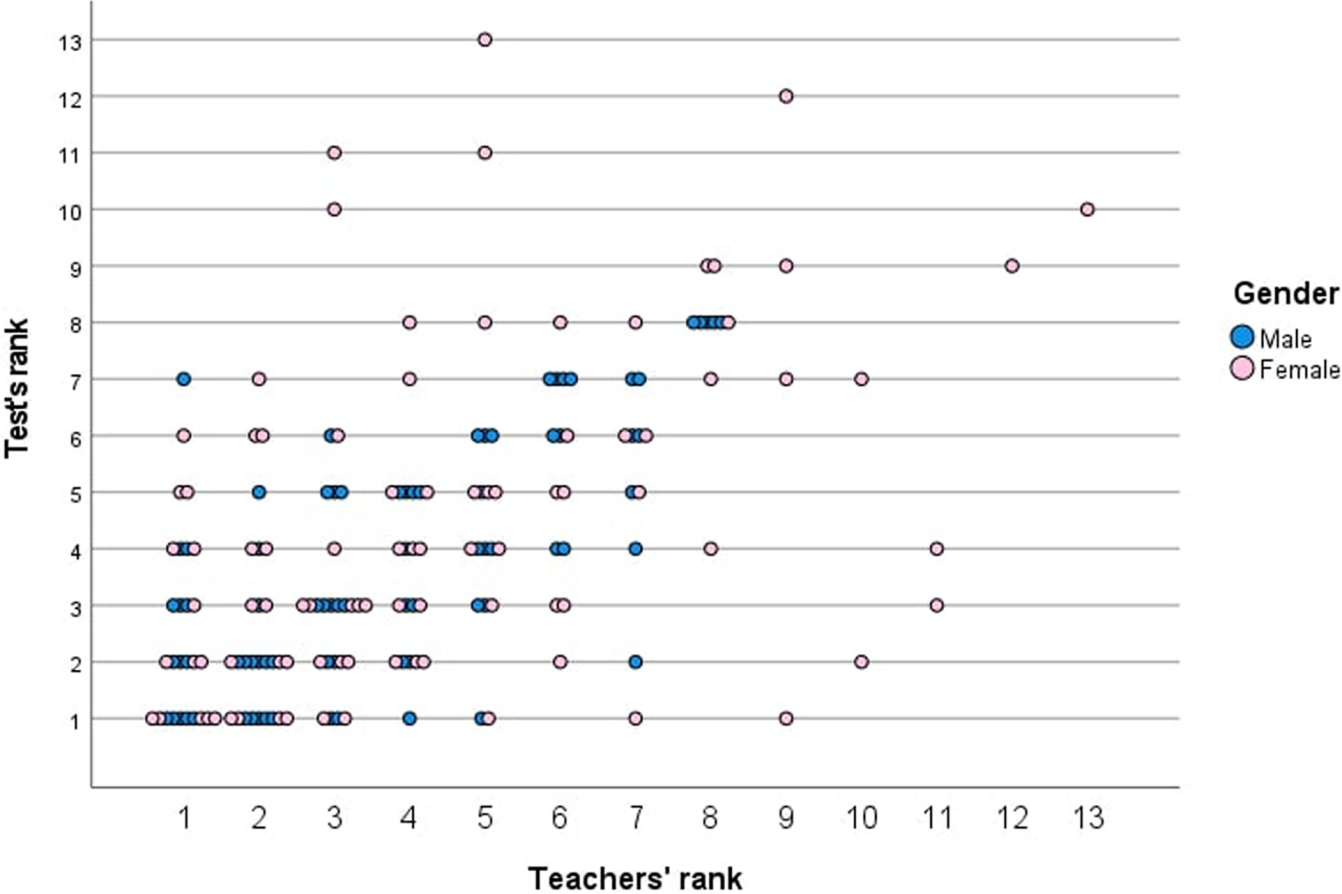
Scatterplot of the correlation between test rank and teacher's rank divided by sex.

Figure 2 shows the correlation between the ranks based on the total test score and the teacher's ranking. The scatterplot does not indicate a particularly high correlation between the two ranks for boys but does indicate a rather low correlation for girls, as confirmed by the Kendall tau-b test, which revealed a correlation of .538 (*p* < .001) for boys and.360 (*p* < .001) for girls.

Last, Table 4 shows the *M* and *SD* in age on the test day according to different age-based categorizations, namely age cohort (i.e., grade) and absolute age on the test day; whereas the first categorizes children born in the same calendar year in the same group, the second categorizes children according to their age on the day when they were tested (6 years = 6.01–6.99 years). Unsurprisingly, the average age of all age groups when categorized as age cohort was significantly higher than when categorized as absolute age, as detailed in Table 4. Furthermore, the distribution of children into the respective age categories was altered. Categorizing by age in our study was based on class in school; for example, 41 children were considered to be 6 years old, whereas only 31 children were considered to be 6 years old when their chronological age determined the age category. In fact, every age category had a different average age when the categorization procedure was changed, and no age group contained the same number of children across procedures. All differences were significant (Table 4).

**Table 4:** Mean (*M*) and standard deviation (*SD*) of children by age on the test day based on age group (i.e., 6–10 years old) and absolute age (i.e., 6–11 years old), with the relative *p* value and 95% confidence interval (CI) of the mean difference.

Age group	*n*	Age at test		Absolute age	*n*	Age at test		*p*	95% CI
		*M*	*SD*			*M*	*SD*		
6	41	6.70	0.30	6	31	6.56	0.20	.028	0.02, 0.26
7	30	7.73	0.27	7	35	7.49	0.32	.002	0.09, 0.39
8	26	8.78	0.27	8	25	8.56	0.29	.007	0.06, 0.38
9	42	9.77	0.28	9	38	9.57	0.27	.002	0.08, 0.32
10	31	10.75	0.30	10	33	10.47	0.29	<.001	0.13, 0.43
11	–	–	–	11	8	11.13	0.06	–	–

## 4 Discussion

The results show that test scores generally increased with age and more steeply among boys than girls. Boys also outperformed girls on most items, especially in the oldest age groups. Meanwhile, children in Italy performed similarly to children in Norway but outperformed ones in Lithuania on nearly every item (25, 30). However, the correlation between teachers’ predictions and the actual test results for boys was low (.538, *p* < .001) and even lower for girls (.360, *p* < .001). The data additionally revealed similar results in PF between the three countries, albeit with differences for some individual items (25, 30). Last, we found that using different procedures for categorizing age affected the results significantly and produced different results between age groups.

As expected, PF, indicated by average total scores, increased with age. Boys generally outperformed girls, mostly on explosive strength and cardiorespiratory fitness, which reflects trends found in samples from Norway and Lithuania (25, 30). PF reference standards for children in Europe show that older children perform even better than younger ones on PF test items, when they involve speed and lower- and/or upper-limb strength (36), which aligns with processes of growth, maturation, and motor development (37). In our study, despite scores that generally increased with age, individual variability was considerable, especially among 10-year-olds. Whereas 6-year-old boys and girls showed highly similar results, among 10-year-olds boys significantly outperformed girls (*p* = .039, 95% CI: −0.695, −0.019). That finding might be explained by girls’ low participation in vigorous PA compared with boys (38), a difference that increases with age. For example, a 50% decrease in PA levels among girls vs. boys 6.5–9.5 years old was reported in 1998 (39). Moreover, in a 2012 study, boys showed greater explosive strength than girls in both upper and lower limbs (40), which may explain why they outperformed girls in our study on all items apart from hopping on one foot, hopping on two feet, and climbing the wall bars. In a 2000 study, the habitual PA level of 2,379 girls also declined by 83% from the ages of 9–10 years to 18–19 years (41), while in 2001 a significant decline in PA was observed from 10 to 16 years of age, when children in general but especially girls were found to spend 75.5% of their days in inactive routines (42).

The performance of children in Italy vs. in other European countries assessed using the same test shows some general trends despite our inability to make statistical comparisons with other countries. First, 4–12-year-old children in Norway slightly outscored their peers in Italy overall due to the age difference, for the sample from Norway included 11- and 12-year-olds whereas Italy's did not (25). The two samples were rather similar regarding anthropometric measures for age groups spanning 6–9 years, whereas 10-year-olds differed considerably. On average, children in Italy were taller (*MD* = 6.3 cm, 4.3%) and heavier (*MD* = 10.8 kg, 24.4%) and had a higher body mass index (BMI; *MD* = 3.6, 17.7%) than their peers in Norway (25). Children in both Italy and Norway showed incrementally higher final scores with increasing age, which fully aligned with expectations based on knowledge about children's growth. On average, children in Norway performed better in throwing a tennis ball, in climbing wall bars, and on the reduced Cooper test, whereas ones in Italy performed better in the standing broad jump and shuttle run. Those results may indicate a difference in musculoskeletal fitness between the two countries, manifested as superior upper-limb strength among children in Norway and, by contrast, superior lower-limb strength among children in Italy (25).

Similar to children in Italy and Norway, older children in Lithuania performed better (30). On individual items, children in Italy of both sexes performed better across all age groups except in throwing a tennis ball (i.e., among boys), climbing the wall bars, and performing the reduced Cooper test. Children in Italy also demonstrated better overall upper- and lower-limb musculoskeletal fitness than ones in Lithuania (30). Moreover, apart from climbing the wall bars and the reduced Cooper test, all average results among 6-year-olds in Italy were better than for ones in Lithuania, even if the anthropometric measures of weight for the age group were the same and with children in Italy being taller by only 1 cm on average (30). Those results encourage further investigations on participation in PA among preschoolers in Italy vs. Lithuania. Again, 9-year-olds in Italy performed better on all test items, except for climbing the wall bars (i.e., for boys) and the reduced Cooper test (i.e., for girls); however, 10-year-olds of both sexes performed very similarly between the countries (30). As before, 9-year-old children in Italy were taller (*MD* = 2.5 cm, 1.8%) and weighed more (*MD* = 2.58 kg, 8.1%) on average, which may explain those results. Despite similar performances on nearly all items, 10-year-olds in Lithuania outperformed ones in Italy in throwing a tennis ball and on the reduced Cooper test (30). The differences in the age group's BMI could explain the differences on the reduced Cooper test; indeed, 10-year-olds in Italy had a far higher average BMI (i.e., 20.3) than ones in Lithuania (i.e., 17.8) (30). However, the same anthropometric values cannot explain differences in throwing a tennis ball, because children in Italy did not seem to benefit specifically from their superior stature.

Meanwhile, children in Norway and Lithuania outperformed children in Italy on the reduced Cooper test. Those results align with past findings comparing the performance of children from 37 countries using the Léger test (16) to assess maximal aerobic power (17). In that study, children in Italy were among the worst performers, whereas children in northern European countries were among the best. As with the reduced Cooper test, climbing the wall bars and throwing a tennis ball were performed better by children in both Lithuania and Norway than ones in Italy, possibly owing to the former's superior upper-limb strength and/or coordination. In either case, the results recommend more cross-national studies on children's physical fitness. Those studies should include more complex, and multidimensional, variables in addition to the usual measuring of strength and endurance.

Although Italy was the European country with the highest prevalence of obese and overweight children less than 10 years old in 2014 (43), the mentioned comparisons revealed rather slight differences in PF possibly because we included children from Cadore, Veneto, a region with particular cultural importance considering PA. In 2021, Veneto ranked among Italy's most physically active regions, one where 31.4% of people more than 3 years old practice sports regularly (44). Therein, Cadore is not a city but an agglomerate of small villages within the Dolomites where children are free to explore and experiment by immersing themselves in the natural environment. Because ample green spaces, woods, and fields are available for engaging in PA and pollution and car traffic are extremely low, parents allow their children to play outside independently at a young age. Moreover, the proximity to Cortina d’Ampezzo and Val Comelico and access to Olympic facilities also influence local participation in winter sports, and the region indeed produces many elite athletes.

Despite the major general influence of sports in Veneto and across Italy, the country has no specific academic curriculum for PE in primary schools (21). That circumstance prompted our second research question, for which we asked teachers to rank students in their class based on the children's perceived PF. Interestingly, the correlation with the ranking from the test results was low for girls (.360) and not high for boys (.538), although the top-three rankings almost always matched exactly, especially for boys. Because some classes had few children, ranking them was easy; however, the correlation in larger classes was sometimes extremely low, especially when more than 10 children had to be ranked. By comparison, the higher correlations between the rankings of 10 boys (i.e., .90) and 10 girls (i.e., .93) assessed by one teacher in Norway may be because Norway requires specific qualifications for PE teachers in primary schools (25, 45).

Although Italy's national guidelines promote the harmonious development of the body through PA, which motivates experimentation with sports, develops self-knowledge of the body, teaches postural habits, and allows discovering different contexts for rhythm, dance, and music (46), teachers in Italy lack specialized training in PE. Furthermore, gyms typically lack the necessary equipment, and the limited curricular time dedicated to PE makes the abovementioned ambitious goals difficult to reach (21). As also mentioned, PF was rather similar on average among children in Italy vs. Norway, possibly given the region in Italy studied, where participation in PA is widespread. Because teachers in primary schools in Lithuania teach pupils all subjects (47), the absence of teachers specifically qualified in PE may also explain differences in PF across age groups and sexes vs. children in northern Italy and Norway. Indeed, to guarantee 9- and 10-year-old children a sufficient level of PF, a 2021 study highlighted the importance of having qualified PE teachers in Lithuania's primary schools and emphasized how schools may play a key role in that effort (48).

In data analysis, we also made a potentially important observation about categorizing children into age groups. In studies with schoolchildren, every child's age has typically been set as that of their age cohort, such that all first-graders are categorized as being 6 years old, second-graders as being 7 years old, and so on, or else their absolute chronological age has been applied (e.g., a child is 6 years old until their 7th birthday). Procedures of categorization can affect the average results for each age group and thus make comparisons difficult across different categorizations and, in turn, different studies. Table 4 shows how different categorizations of children in our study might have altered the average age and our results. All ages based on absolute chronological age were significantly higher than when based on age cohort, as detailed in Table 4. A third possible categorization involves using chronological age on the test day, which may again produce different results. In fact, two children in the same age cohort may be a year apart in chronological age. That categorization based on age cohort explains the relative age effect (49), which favors children born earlier in the calendar year because they are bigger and more developed on average (50). Such older children have been shown, for example, to receive better grades in PE (51), and the relative age effect also greatly affects scores of PF measured among children (52).

Fjørtoft et al.'s article introducing the test that we used neither specifies how they categorized age nor advises how it should be categorized (25), and the same is generally true in studies examining children in age groups (38, 53, 54). Because comparing results across studies is difficult even when the same test or instrument is used, all studies on children that categorize them by age should do so according to the same criteria, or at least specify which criteria for categorization were applied, in order to prevent the misinterpretation of results. Considering such potential differences in age categorization, it is also uncertain whether published comparisons are reliable, including international ones.

Some limitations of our findings warrant mention. First, our study's sample may not represent Italy's population, because data were collected in a selected area in northeastern Italy, where a different cultural and social environment could have affected the results. Second, statistically comparing children in Italy with children in Norway and Lithuania was impossible, because the age groups studied did not align. In fact, no studies conducted in Norway have distinguished age groups but have instead presented overall results for 4–12-year-olds (25), 5–6-year-olds (27), 4–6-year-olds (28), 9- and 12-year-olds (29), and 11- and 12-year-olds (26). Third, each subgroup included a small number of children, and any between-group differences (i.e., by age or sex) in the data from Italy should be interpreted accordingly. Moreover, many children in our study did not perform the wall-bar climbing task, which also colored our results. The item nevertheless remained in the data set and factored into the total score, because 52 children did in fact perform the item, and their results did not differ much from the children from other countries, similar to all other items. In that light, the results for the wall-bar climbing task are probably as reliable as the results for the other items and should not have significantly affected the total score. Even so, we cannot compare results for that item for any smaller groups, including age- or sex-based groups, or compare the performance on the wall-bar climbing task between countries. The reader should also be aware that the test results from other European countries that we compared with our results were from studies conducted a few years ago, which might have affected our results had relative between-country changes in PF occurred during the interval between the results. That problem is common when comparing results between different studies, including review studies, or when data are pooled, as in meta-analyses and in large databases.

Further investigations need to pinpoint potential differences in PF between children in southern and northern Europe depending on age and sex, especially in relation to cultural and social differences. Researchers also need to identify potential differences in PF at primary schools with and without qualified PE teachers. After all, the limited ability of current teachers to assess children's PF could affect PF, and the results could also inform debates, for example, about new national regulations in Italy. Last, researchers examining children's PF should clarify how they have categorized age so that results from the same test batteries can be compared.

## 5 Conclusions

The scores on the PF test for children in northeastern Italy were similar to scores reported for children from other European countries who had taken the same test. Thus, there seems to be little difference between the PF of children in Italy, at least ones from the northeastern region Cadore, and the PF of children in several other European countries.

Teachers’ assessment of their students’ PF in Italy did not correlate particularly well with actual test scores, especially for girls. Although that finding is interesting insofar as PE is important for promoting PA and thus increasing children's PF levels, it cannot be taken as direct support for the regulation that teachers need to have education in PE in order to teach PE. It might nevertheless signal that teachers in Italy, at least those who participated in our study, have a slightly different perception of PF than what the test measures.

Our study’s results also indicate that research reporting results from tests comparing children in different age cohorts or from studies on the development of PF in a population over time should describe how the children were categorized by age, for the results may vary depending on the different categorization procedures and may thus make comparisons unreliable.

## Data Availability

The original contributions presented in the study are included in the article/Supplementary Material, further inquiries can be directed to the corresponding author.
